# Outcomes and associated factors of open abdomen after urgent laparotomy at a University Hospital in Southern Brazil: a retrospective study

**DOI:** 10.1590/0100-6991e-20243653-en

**Published:** 2024-07-26

**Authors:** ARIANA IEDA LIMA FERREIRA DA SILVA, IMAD SHEHADEH, EDUARDO FALCO KNAUT, ENRICO PRAJIANTE BERTOLINO, CARLOS EDMUNDO RODRIGUES FONTES

**Affiliations:** 1 - Hospital Universitário Regional de Maringá, Departamento de Cirurgia Geral - Maringá - PR - Brasil; 2 - Universidade Estadual de Maringá, Departamento de Medicina - Maringá - PR - Brasil; 3 - Universidade Estadual de Maringá, Programa de Mestrado em Gestão Tecnologia e Inovação em Urgência e Emergência - Maringá - PR - Brasil

**Keywords:** Open Abdomen Techniques, Laparotomy, Abdomen, Acute, Negative-Pressure Wound Therapy, Técnicas de Abdome Aberto, Laparotomia, Abdome Agudo, Tratamento de Ferimentos com Pressão Negativa

## Abstract

**Introduction:**

The technique of open abdomen refers to a surgical procedure that intentionally involves leaving an opening in the abdominal wall. This study aimed to evaluate the clinical outcomes, mortality, and morbidity of patients undergoing open abdomen in a public hospital in Brazil and investigate associated risk factors associated with the outcome.

**Methods:**

Data from electronic medical records were collected from 2017 to 2022. The variables were used for descriptive analyses, association analysis, and survival analysis using the Kaplan-Meier curve.

**Results:**

The sample included 104 patients, with 84 presenting with acute abdomen and 20 with trauma, having highly variable ages and comorbidities. Peritonitis and the need for early reoperation were the most common indication for the procedure, each accounting for 34%, and negative pressure wound therapy was the most commonly used technique. Fistula was the most frequent complication, with the majority forming in the early days after the surgery. The number of interventions and open abdomen time obtained statistical significance in comparison with the outcome. The overall mortality rate was 62,5%.

**Conclusion:**

Despite open abdomen being a technique that can have benefits in controlling intraabdominal contamination and preventing abdominal compartment syndrome, its implementation is associated with complications. The mortality and complication rates were high in this sample. The decision to use the technique should be individualized and based on several factors, including the indications and the patient’s clinical status.

## RESUMO

**Introdução:**

A técnica de abdome aberto refere-se a um procedimento cirúrgico que envolve deixar deliberadamente uma abertura na parede abdominal. Este estudo teve como objetivo avaliar o desfecho clínico, mortalidade e morbidade de pacientes submetidos ao abdome aberto em um hospital público do Brasil e investigar fatores de risco associados ao desfecho.

**Métodos:**

Dados a partir de prontuários eletrônicos foram coletados de 2017 a 2022. As variáveis foram utilizadas para análises descritivas, análise de associação e de sobrevivência pela curva Kaplan-Meier.

**Resultados:**

A amostra correspondeu a 104 pacientes, sendo 84 por abdome agudo e 20 por trauma, com idade e número de comorbidades variados. A peritonite e a necessidade de reabordagem precoce foram as causas mais comuns para a indicação do procedimento, 34% cada, e a terapia de pressão negativa foi a mais utilizada neste estudo, seguindo a técnica de Barker. Fístula é a complicação mais frequente (41%), sendo que a maioria se formou nos primeiros dias após a realização da cirurgia. O número de intervenções e o tempo de abdome aberto obtiveram significância estatística na comparação com o desfecho. A mortalidade geral foi de 62,5%.

**Conclusão:**

Apesar de o abdome aberto ser uma técnica que pode trazer benefícios no controle da contaminação intra-abdominal e prevenção de síndrome compartimental abdominal, sua realização está associada a complicações. A taxa de mortalidade e morbidade foram elevadas nesta amostra. A decisão para uso da técnica deve ser individualizada e baseada em vários fatores, incluindo as indicações e o estado clínico do paciente.

## INTRODUCTION

The technique known as open abdomen (OA) or peritoneostomy refers to a surgical procedure that involves deliberately leaving an opening in the abdominal wall at the end of an intra-abdominal intervention^1^ ^-^ ^3^.

This technique is often used in cases of abdominal trauma, intra-abdominal sepsis, the need for early reapproach (second look), and other conditions that can cause intra-abdominal hypertension. Although it is relatively simple, an open abdomen can be associated with complications, such as infection, hemorrhage, and fistula formation. Peritonitis is one of the main indications for the procedure, and can be caused by different etiologies, such as intestinal perforation, perforated diverticulitis, perforated appendicitis, necrotizing pancreatitis, among others¹.

Several factors can influence the occurrence of complications after the open abdomen, such as the surgical technique used, the duration of the procedure, the presence of comorbidities, and the length of time the peritoneum remains opened. Some studies suggest that the duration of the open cavity may be related to the occurrence of complications, especially the formation of fistulas^4^. The use of open abdomen in cases of peritonitis may allow the control of infection, the washing and debridement of the abdominal cavity, and the prevention of abdominal compartment syndrome. However, intestinal fistula is a potentially serious complication that can occur in patients after laparotomy, especially with loop manipulation¹.

The overall objective of the study was to evaluate the clinical outcomes, morbidity, and mortality of patients undergoing open abdomen (OA) after emergency laparotomy at a university hospital in Southern Brazil, and to investigate the risk factors associated with the outcome.

## METHODS

### Study design and location

This is a cross-sectional, retrospective, and descriptive study, which enrolled all patients undergoing emergency operations from January 2017 to December 2021. 

The Regional University Hospital of Maringá (HUM) is a reference center for more than 115 municipalities in the Northwest macro-region of the state of Paraná, serving a population of approximately two million. Among these, acute abdomen and trauma stand out.

### Inclusion and exclusion criteria

We selected patients based on the type of procedure (exploratory laparotomy) described in the operating room surgical report and collected the data from the electronic medical records. We included all patients who had temporary closure of the abdominal cavity after urgent exploratory laparotomy, regardless of the technique used to perform this closure. We excluded patients whose data in the hospital record and in the medical archives were insufficient, which corresponded to only two cases. 

The clinical variables analyzed were age, sex, American Society of Anesthesiology (ASA) classification, surgery indication, surgical technique, time to reapproach, number of approaches, period of open cavity, complications, method of abdominal wall closure, outcome, and clinical and surgical complications.

### Data analysis

We tabulated and organized the data using Excel.

Descriptive analysis was the first stage, consisting of the presentation of the collected data using descriptive measures, such as mean, median, standard deviation, and frequency. Then, we performed hypothesis tests to evaluate the statistical significance of the differences observed between groups, such as non-parametric ones, the Pearson’s chi-square test, and the Fisher’s exact test.

In addition, we evaluated the relationship between the variables using Pearson’s and Spearman’s correlation coefficients. We calculated the Odds Ratio (OR) and the 95% confidence interval (CI) for comparisons between the groups.

We used survival analysis with the Kaplan-Meier method to evaluate the outcome (discharge or death). We also performed sensitivity analyses to assess the robustness of the results obtained, including subgroup testing and the exclusion of extreme observations. All statistical analyses were performed with the Python software.

The project was approved by the Committee for the Regulation of Academic Activities (COREA) of HURM (n°: 059/2020) and by the Permanent Committee on Ethics in Research with Human Beings of the State University of Maringá (COPEP/UEM) - (CAAE: 63638822.4.0000.0104).

## RESULTS

### Patients’ sociodemographic and clinical characteristics

There were 1,219 emergency laparotomies between January 2018 and December 2022, of which 104 resulted in OA (8.5%), 84 (80.8%) due to acute abdomen and 20 (19.2%) due to abdominal trauma.

The mean age of the patients was 55.77 years, and the median, 64 years. This suggests that the age distribution is not symmetrical and may be skewed to the left; most patients are over 50 years of age. The standard deviation was 20.08 years, which indicates that the dispersion of age is relatively large. In addition, the first quartile (25%) is 38 years old, and the third quartile (75%), 72 years old. The age range is 77 years, from 15 to 92.

[Fig f1]

**Figure 1 f1:**
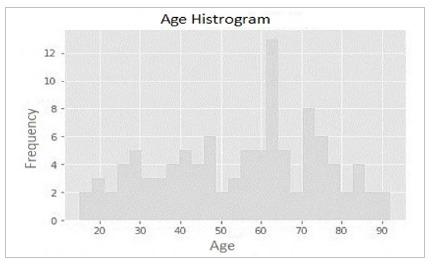
Histogram of patients’ age distribution.

Most patients in the sample were male, representing 79%, while females represent 21%.

Of the studied patients, 65 (62.5%) were classified as ASA I and II, while 39 (37.5%) were classified as ASA III or higher.

In this data set, 76 patients had comorbidities, which represents 73.1% of the total, while the other 28 patients (26.9%) were previously healthy. The most common were systemic arterial hypertension (SAH), smoking, alcoholism, type-2 diabetes mellitus, and previous stroke.

### Factors related to the initial cause and indication of OA

[Table t1]

**Table 1 t1:** OA percentiles according to cause.

Initial Cause	Relative Frequency
Acute Inflammatory Abdomen	28,8%
Acute Obstructive Abdomen	22,1%
Acute Perforative Abdomen	19,2%
Acute Vascular Abdomen	10,6%
Open Abdominal Trauma	10,6%
Blunt Abdominal Trauma	8,7%

Of the 104 patients, the number of indications included 36 (34%) due to peritonitis, 36 (34%) due to the second evaluation, 24 (22.6%) due to hemodynamic instability, and 10 (9.4%) due to abdominal compartment syndrome.

The type of OA performed in this study was predominantly the negative pressure dressing (NPT), in 96 cases, improvised with make-do materials from the operating room. Only 7.8% corresponded to the temporary closure with the Bogotá bag (8 cases). 

### Medical and surgical complications

The descriptive analysis revealed that 43 patients had fistula, the most frequent complication. On the other hand, 41% of patients had no complications, also resulting in 43 cases. In addition, 16% of patients had surgical wound infection. The minority had retained hematoma, intra-abdominal abscess, seroma, ischemia of loops and extremities, adhesions, evisceration, and abdominal and wall bleeding.

There were no surgical complications in 41.35% of patients, while 58.65% had at least one. The mean OA time was 2.49 days, with median of one day. The standard deviation of time was 3.12 days. As for the relationship between time and complications, patients who had surgical complications remained with OA for a mean period of 3.24 days, while those without complications had a mean time of 1.60 days. This difference in means was statistically significant (p = 0.0001). However, there was no clear correlation between the duration in days and the number of surgical complications, as patients with different times may have had the same number of complications.

### Enteric fistula

Most fistulas formed in the first few days after the first surgery, the third day displaying the highest number of cases. Some patients presented with fistulas later, and day 39 was the latest. 

The standard deviation is approximately 23 days, and the median is five days. The coefficient of variation translates the dispersion of the data relative to the mean, and the higher the coefficient, the greater the data dispersion. In this case, the coefficient of variation is about 105%, which indicates considerable dispersion.

### Clinical complications

Clinical complications occurred in 60.6%. The five most common complications were sepsis (31.7%), acute renal failure (28.8%), pneumonia (24%), reversed cardiopulmonary arrest (18.3%), and delirium (16.3%). On the other hand, 39.4% of the patients had no complications. 

### Patient outcomes after emergency laparotomy

Of the 104 patients, 65 died, equivalent to 62.5% of the sample. The mean age was 59.55 and the standard deviation was 19.26. Of all, 31 were discharged, equivalent to 29.8% of the sample. The remaining were transferred to another service.

[Table t2]

**Table 2 t2:** Patients with or without fistula and open abdomen time

	No fistula	With fistula
Number of cases	61	43
Average OA time (days)	2,81	3,63
Confidence interval (95%)	2,46-3,16	3,20-4,05
p-value	< 0,001	

[Fig f2]

**Figure 2 f2:**
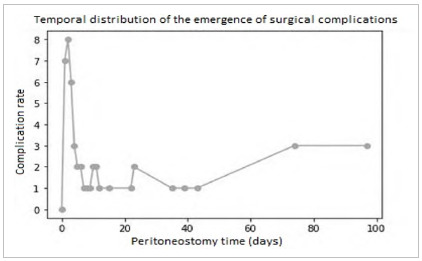
Complications and time of open abdomen.

### Survival analysis

Based on the data provided, we performed a survival analysis using the Kaplan-Meier method to assess mortality in relation to the length of open abdomen.

[Fig f3]

**Figure 3 f3:**
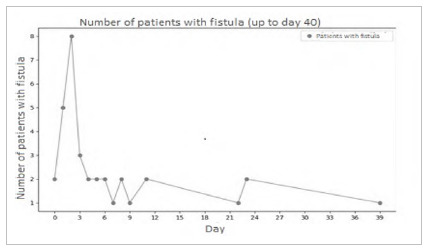
Fistula formation according to the day after the initial surgery.

The following graph shows the probability of survival over time. Mortality was high in the first few days, with a significant drop in the likelihood of survival within the first two days. From then on, mortality gradually decreases but remains relatively high in the first 10 days, with a steeper drop.

Most patients (about 60%) died within the first 10 days. The probability of survival at 30 days was about 37%, while at 60 days it was about 24%. There was a plateau effect after a high mortality rate in the first three weeks.

This chart can help in clinical decision-making to assess the risk of mortality in relation to OA duration.

### Factors associated with outcome

We excluded eight patients due to transfer or dropout.

**Figure 4:**
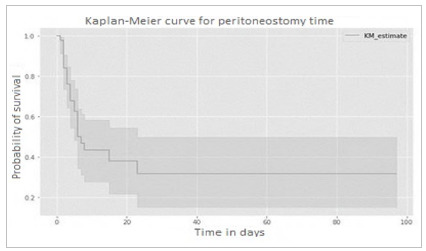
Kaplan-Meier curve of survival in relation to open abdomen time..

### Open abdomen technique

The data provided describe the outcome of different abdominal surgery techniques in relation to the number of patients who died or were discharged from hospital. Of the 82 patients who underwent NPT, 53 (64.6%) died, while 29 (35.4%) were discharged. In the other technique, 11 of the 15 patients (73.3%) died and four (26.7%) were discharged.

Note that the Odds Ratio (OR) value for the vacuum-open abdomen is equal to 1, as it is the reference for comparison with other surgical techniques. The OR value for the Bogotá bag is 2.44. 

The chi-square value was 2.165, with a degree of freedom of 1, and a p-value of 0.1418, indicating insufficient evidence to reject the null hypothesis that there is no statistically significant difference in the proportions of deaths between the two surgical techniques. The confidence interval for the OR was 0.579 to 1.764, indicating that we cannot be sure that there is a statistically significant difference in the odds of mortality.

### Number of interventions

The data analyzed included 96 patients, of whom 77 resulted in death and only 19 were discharged. Regarding the number of approaches, the mean was 5.38, with a standard deviation of 4.47, ranging from one to 19 approaches.

[Table t3]

**Table 3 t3:** Quantification of variables and the outcome.

NPT	Odds Ratio	Chi-square	p-value	95% CI
Bogotá	1			
ASA 1/2	2.44	2.165	1.418	0.579-1.764
ASA ≥ 3	1			
Age < 60	5.58	12.5	4	1.92-19.27
Age ≥ 60	1			
With comorbidity	6.71	11.2	8	2.43-18.56
No comorbidity	5.29	42.87	<0.001	2.96-9.48
OA in the 1st surgery	-	-	-	-
OA after the 1st surgery	15.867	70.837	< 0.001	7.210-34.968
AA após a 1° cirurgia	6.75	6.184	0.013	1.534-29.721

When analyzing the proportions of deaths and discharges, we observed that most patients died (80.2%), while only 19.8% were discharged. 

A very low p-value, less than 0.001, indicates that the association is highly unlikely to be due to chance. The confidence interval, from 0.008 to 0.096, suggests that the true odds ratio (in the general population) is most likely in this range. 

### Open Abdomen Time

The dataset consisted of 96 patients, with open abdomen time varying every 10 days, by way of categorization. The chi-square test showed a significant association between time and outcome (p < 0.001). More specifically, patients with a longer OA time had a higher mortality rate compared with those with a shorter time.

The chi-square test for the subset of patients with a time of up to 10 days also showed a significant association with the outcome (p = 0.022). Again, the mortality rate was higher in the longer-term group.

## DISCUSSION

In this series, 8.5% of emergency laparotomies over a five-year period resulted in an open abdomen. We observed that the age distribution is relatively normal, with a slight skewness to the left, which suggests that a slightly higher number of older patients, with most ages distributed between 60 and 70 years. Notably, there is a significant number of younger patients (under 40 years) and some older ones (over 80 years).

There was predominance of causes of acute abdomen typical of older patients, with more comorbidities and worse ASA scores. Different etiologies are included in this group, such as diverticulitis, appendicitis, adhesions, intestinal lumen stenosis due to tumor, volvulus, hernias, ulcers, among others. Again, more frequent causes in men. The chance of death in individuals older than 60 years of age is 6.71 times higher. If one has at least one comorbidity, the risk of death increases by 5.29 times. In this same vein, death is 5.6 times more likely to happen in ASA 3 or higher patients. All these facts allow us to draw a profile of the sample, knowing who in general is not a good candidate to survive OA and, in summary, the analysis shows that patients with comorbidities have a significantly higher risk of dying compared with patients without comorbidities.

The criteria for indicating the open abdomen technique include refractory shock, abdominal hypertension, organ failure, refractory hypothermia, abdominal wall detachment, intra-abdominal infection, abdominal compartment syndrome, and inability to close the abdominal wall primarily^1^ ^,^ ^3^. However, the decision to use the technique should be individualized and based on several factors, including the reason for the indication and the patient’s clinical status^5^.

On several occasions, we could not find a properly clarified OA indication in the medical records of the studied patients. In 2018, Coccolini et al. published guidelines for the indication of temporary closure of the abdomen, with the degrees of recommendation regarding the methods for the optimal management of the open abdomen^6^.

More than 85% of patients with an open abdomen due to inflammatory causes died, peritonitis being the main indication for the technique. With a mortality rate of 44%, not only the acute inflammatory abdomen causes peritonitis. Also with the same indication rate, approximately 74% of second look patients survived. In the same pattern, 76% of the cases due to hemodynamic instability had their shock cause controlled. It is important to highlight that these data only point to an association between the different indications and mortality rates, and that other factors may also influence outcome. In addition, it is necessary to consider that mortality is not the only measure of success or failure of a surgical procedure, and other clinical outcomes should also be evaluated.

In this study, as well as in the literature, the most used technique for temporary closure of the abdomen was negative pressure wound therapy, corresponding to 83% of the cases. It is noteworthy that the local hospital service does not have access to specific commercial dressings for the negative pressure technique, which led surgeons to use improvised materials, constituting the Barker technique^7^. Despite these limitations, the improvised wound negative pressure technique has been effective and safe, with results like those found in studies that used commercial negative pressure techniques^7^ ^,^ ^8^. However, further research is needed to evaluate the efficacy and safety of these improvised techniques in a larger sample of patients. In the general evaluation, mortality was not influenced by the technique, and 64.6% of the patients with vacuum dressings died, compared with 73.3% with the Bogotá bag.

Two societies promoted the International Register of Open Abdomen (IROA), with the dissemination of results on the use of the open abdomen technique as of 2017^9^. With participants from hundreds of countries for more than one year, 369 adult patients were registered. The indications are consistent with those mentioned above, and the most common method was NPT. Complications occurred in 38% (with a positive linear correlation of days until wall closure), 10.5% due to fistulas, and 17.2% mortality. NPT has been superior for cases of peritonitis, and Bogotá bag or skin closure, for trauma.

According to the study conducted by the IROA group^9^, negative pressure dressing is the most common option (46.8%) for temporary abdominal closure, due to its ability to facilitate the formation of wound granulation, prevent the formation of fistulas, and reduce wound contamination^6^. Atema^14^ found that this dressing associated with continuous fascial traction produced better outcomes in terms of primary fascial closure of the abdomen, although it presented a higher risk of fistula formation. A systematic review^8^ published in 2022 concluded, based on the available data from clinical trials, that it was not possible to state with certainty whether negative pressure therapy has any benefit in primary closure of the abdomen, in the occurrence of adverse events (such as fistula formation), in overall mortality, or in the length of hospital stay when compared with the Bogotá bag. More research is needed to evaluate these results.

The use of open abdomen in cases of severe peritonitis is a controversial topic, with advantages and disadvantages that must be carefully evaluated. Among the advantages are the possibility of better control of intra-abdominal contamination and the prevention of abdominal compartment syndrome, which can lead to multiple organ failure. On the other hand, the technique may be associated with complications such as fistulas, surgical wound infections, bleeding, and dehydration9. Moreover, the length of open abdomen may be related to an increase in mortality and morbidity^12^ ^,^ ^13^.

The relationship between time and complications is an important topic in clinical practice. Studies suggest that longer duration is associated with a higher risk of complications, including wound infection, fistula development, and dehydration^6^ ^,^ ^14^ ^,^ ^15^. For example, an observational study of 77 patients with abdominal compartment syndrome treated with open abdomen showed that the mean time was significantly longer in patients who developed complications compared with those without them (p = 0.009)¹¹. Another retrospective cohort study with 54 patients who underwent laparotomy followed by an open abdomen showed that a prolonged time was an independent risk factor for the development of enteric fistula^13^. 

There was occurrence of fistula in the 43 cases in this study. Most patients (29.8%) had fistula after the first approach, and this rate decreased as the number of approaches increased, with a more pronounced decrease after the third procedure. This suggests that the surgeon’s experience in indicating a new intervention is an important factor in the prevention of this complication.

The incidence of enteroatmospheric fistula varies in the literature, from 4.5% to 25% in the open abdomen due to trauma, and from 5.7% to 17.2% in non-traumatic laparotomies^11^ ^,^ ^15^. In this sample, we observed a higher frequency of fistulas, though we should highlight that the number is limited and biased towards the characteristics and types of care of local patients.

The data suggest early fistula formation, as is the case in most of the current literature. The mean time of open abdomen is two days longer in patients with fistulas compared with other complications. We should note that the time difference between patients with and without complications was 1.64 days, which was statistically significant, but not related to the number of complications. That is, the longer one has an open abdomen, the greater the risk, although complications tend to happen in the initial days, but it is not possible to say how many complications to expect. Therefore, the decision on OA duration should be made with caution, trying to minimize the time of exposure of the internal organs. 

It is important to note that the data presented do not include information on the time elapsed between the first surgery and the development of the fistula, or the comparison with other relevant clinical information, such as the indication or patients’ comorbidities. 

Clinical complications were also present in large quantities. As mentioned, peritonitis prevailed, sepsis of abdominal focus being the most common (31.7%). This was followed by acute renal failure (28.8%), which is intrinsically related to the state of shock in which the patients are, of probable prerenal etiology due to poor perfusion. Finally, another important problem is ventilator-related pneumonia, with about 24%, typical of any prolonged intubation. Approximately 20% of the patients had reversed cardiorespiratory arrest during their stay.

In the literature, mortality rates vary widely, depending on the severity of peritonitis and the presence of other comorbidities. Studies indicate that the mortality of patients with open abdominal peritonitis can be as high as 70%, but this value can be reduced with the appropriate use of infection control techniques and patient monitoring^12^. In agreement with our findings, another study showed that 44% of patients undergoing OA died^3^. Compared with this study (62.5% of deaths), the difference demonstrates an important variability, as well as the multiple influencing factors.

In the same line of reasoning, mortality was closely related to length of stay in an ascending manner, but most deaths occurred within 10 days. The Kaplan-Meier curve (Figure 4) shows high mortality in the initial days, with a plateau from the third week onwards. The log-rank test confirmed that this difference in survival was statistically significant (p < 0.001). The mean age of the dead patients, approximately 60 years, is consistent with the profile of the sample. With a high number of approaches, most of which resulted in death (80.2%), patients who needed to return to the operating room many times benefited, with a decrease in mortality. The OR analysis showed that the number of approaches is significantly associated with the outcome, a higher number being associated with a lower risk of death.

One can assume that the procedures were well indicated, allowing surgeries (usually washing, breaking of adhesions, checking of the abdominal cavity, suturing, among others) to those who really needed them. On the other hand, it is questionable when OA should be performed, and patients placed under the technique from the second surgery onwards show lower mortality. The highest survival rate was of those with faster wall closure. 

The analysis performed after dividing into groups indicates a significant difference in relation to the patients’ outcomes, with a higher percentage of deaths in the group submitted to OA in the first surgery. This association may be related to the severity of the case, in which the patient with the worst prognosis underwent OA for rapid stabilization in an intensive care unit, but there are no data to confirm this statement. 

Importantly, these results are based on a limited sample and should therefore be interpreted with caution. Future studies with larger sample sizes are needed to confirm or reinforce these findings and provide more robust evidence to guide clinical decisions.

## CONCLUSION

The maintenance of open abdomen leads to high mortality, and is associated with advanced age, ASA classification ≥ 3, and the presence of comorbidities. In addition, the need for an open abdomen in the first approach is also associated with a significantly increased risk of negative outcome. 

The number of approaches appears to be associated with an increased risk of fistula. However, open abdomen time does not appear to be a significant risk factor for such a complication. There was no increase in mortality with the greater number of surgical interventions and, on the contrary, chronic patients benefited from the operation when well indicated. NPT was preferred but did not change the outcome significantly.
